# Optical polarization properties of (11–22) semi-polar InGaN LEDs with a wide spectral range

**DOI:** 10.1038/s41598-020-64196-w

**Published:** 2020-04-28

**Authors:** N. Poyiatzis, J. Bai, R. M. Smith, M. Athanasiou, S. Ghataora, T. Wang

**Affiliations:** Department of Electronic and Electrical Engineering, University of Sheffield, Mappin Street, Sheffield, S1 3JD United Kingdom

**Keywords:** Inorganic LEDs, Nanophotonics and plasmonics

## Abstract

Electroluminescence polarization measurements have been performed on a series of semi-polar InGaN light emitting diodes (LEDs) grown on semi-polar (11–22) templates with a high crystal quality. The emission wavelengths of these LEDs cover a wide spectral region from 443 to 555 nm. A systematic study has been carried out in order to investigate the influence of both indium content and injection current on polarization properties, where a clear polarization switching at approximately 470 nm has been observed. The shortest wavelength LED (443 nm) exhibits a positive 0.15 polarization degree, while the longest wavelength LED (555 nm) shows a negative −0.33 polarization degree. All the longer wavelength LEDs with an emission wavelength above 470 nm exhibit negative polarization degrees, and they further demonstrate that the dependence of polarization degree on injection current enhances with increasing emission wavelength. Moreover, the absolute value of the polarization degree decreases with increasing injection current. In contrast, the polarization degree of the 443 nm blue LED remains constant with changing injection current. This discrepancy can be attributed to a significant difference in the density of states (DOS) of the valence subbands.

## Introduction

The past two decades have witnessed the major advancement in developing III-nitride semiconductor based optoelectronics, epitomized by blue InGaN light emitting diodes (LEDs). However, it is worth highlighting that these achievements have been predominantly founded upon *c-plane* substrates, where the polar orientation poses a number of fundamental limits that restrict the great potential to further develop III-nitride optoelectronics. The strong polarization effects are formed as a result of the piezoelectric fields induced by the lattice mismatch of InGaN and GaN, resulting in the quantum-confined Stark effect (QCSE). Consequently, InGaN LEDs grown on *c-plane* substrates suffer a reduced overlap of their electron and hole wavefunctions, which leads to a reduced quantum efficiency^1,2^. This effect increases in longer wavelength LEDs (green or yellow), where higher indium content is required and thus the piezoelectric fields are further enhanced. Eventually, this has resulted in the well-known green-gap crisis. In addition, it is also with great challenges to even incorporate more indium into *c-plane* GaN during growth, but higher indium content InGaN is necessary for these green and yellow wavelength LEDs^3,4^.

Very recently, long wavelength emitters have gained increasing focus due to not only solid-state lighting but also optogenetic applications. In the latter, III-nitride visible emitters with a longer emission wavelength play an important role in manipulating neurons, which will assist in the understanding of neural circuit behaviors and responses under light stimulating conditions. This important work will help in finding a solution to curing Parkinson’s disease, mood disorders and many more^5,6^.

III-nitride growth along a semi-polar or non-polar orientation is an effective approach in overcoming the aforementioned challenges above. Among a number of semi-polar orientations, the (11–22) direction has gained considerable attention in developing long wavelength LEDs due to an enhanced indium incorporation efficiency and reduced QCSE in the InGaN grown on the (11–22) GaN surface^7–9^. Furthermore, semi-polar or non-polar LEDs offer a unique feature that their polar *c-plane* counterparts lack, which is an intrinsic polarized emission. This is owed to their low crystal symmetry, where the inhomogeneous biaxial strain of a semi- or non-polar plane results in the splitting of the uppermost valence bands leading to optical anisotropy and thus polarized emission. A polarized light source plays a vital role in many applications, such as backlighting. The current approach to LED backlighting utilizes a polarization filter to reject unpolarized light, but this suffers ~30% optical energy loss due to the utilization of a polarizing filter^10^. Therefore, if semi-polar or non-polar LEDs are instead used in backlighting, such a polarizing filter becomes unnecessary and at least ~30% wasted energy consumption is saved.

However, two main challenges in developing high-performance semi-polar LEDs need to be overcome. The first is to obtain high-quality semi-polar GaN on industry compatible substrates, such as sapphire. Recently, our team have demonstrated semi-polar InGaN LEDs overgrown on our (11–22) semi-polar GaN templates with significantly improved crystalline quality, leading to high-performance semi-polar InGaN LEDs across a wide spectral range, up to amber^11–13^. Secondly, it is crucial to fully understand the polarization mechanisms of different wavelength semi-polar (11–22) InGaN LEDs operating under a range of different injection currents. Although there have been a number of reports in this field^14–16^, so far these mechanisms are still not well-understood, consequently leading to controversial debates.

It has previously been observed that InGaN/GaN LEDs grown on semi-polar (11–22) bulk substrates operating in the blue, green and amber spectral region do not exhibit any enhancement in polarization degree with increasing indium composition^17^, while a monotonic increase in the absolute value of polarization degree has conversely been reported in semi-polar (11–22) InGaN/GaN LEDs with similar rising indium content^18^. The observable polarization switching phenomena has been attributed to that two distinct top valence subbands approach each other with increasing indium content, eventually leading to a crossover of these at ~30% indium content^19^. However, there still exist some debates surrounding the formation of this mechanism. On the one hand, polarization switching could be caused by large InN deformation potentials, while other studies have concluded that it is due to partial strain relaxation or inhomogeneous strain effects across the (11–22) InGaN quantum wells^20,21^. It has also been reported that a change in polarization degree has been observed when a relaxed underlying InGaN buffer layer is employed prior to the growth of an emitting InGaN quantum well structure, where underlying InGaN buffer layers with different thickness and indium content have been considered^22,23^. Furthermore, a combination of the self-consistent Poisson and 6 × 6 k•p Schrödinger equations has been used to predict the influence of polarization properties as a function of injection current in (11–22) semi-polar LEDs^24^. However, there are not experimental data to support these predictions.

In this work, a systematic study has been conducted on a series of semi-polar InGaN LEDs with a wide range of indium content (covering emission wavelengths from 443 to 555 nm) grown on (11–22) semi-polar GaN templates with a high crystalline quality. Emission polarization has been investigated as a function of both indium content and injection current.

A series of InGaN single quantum well (SQW) LEDs with different indium composition were grown on overgrown semi-polar (11–22) GaN templates on *m-plane* sapphire by metal-organic chemical vapor deposition (MOCVD). The semi-polar (11–22) GaN templates were obtained by means of our well-established overgrowth technique on regularly arrayed micro-rod templates. More details regarding the crystal quality can be found elsewhere, which include x-ray measurements and transmission electron microscopy (TEM) measurements^11–13^.

All the LED structures are similar except for in their indium content (indium content from 0.15 to 0.3 for each case). Each LED structure consists of a 1 µm Si-doped n-type GaN layer, a 4 nm single InGaN quantum well sandwiched between two 9 nm thick un-doped GaN barriers and a final 150 nm p-type GaN capping layer (refer to Fig. 1S in Supplementary Information for scanning electron microscopy (SEM) and TEM characteristics and to Fig. 2S in Supplementary Information for a schematic diagram for the detailed LED structures). By means of a standard photolithography technique and subsequent dry-etching processes, LEDs with a standard 330 ×330 μm^2^ mesa size have then been fabricated.

**Figure 1:**
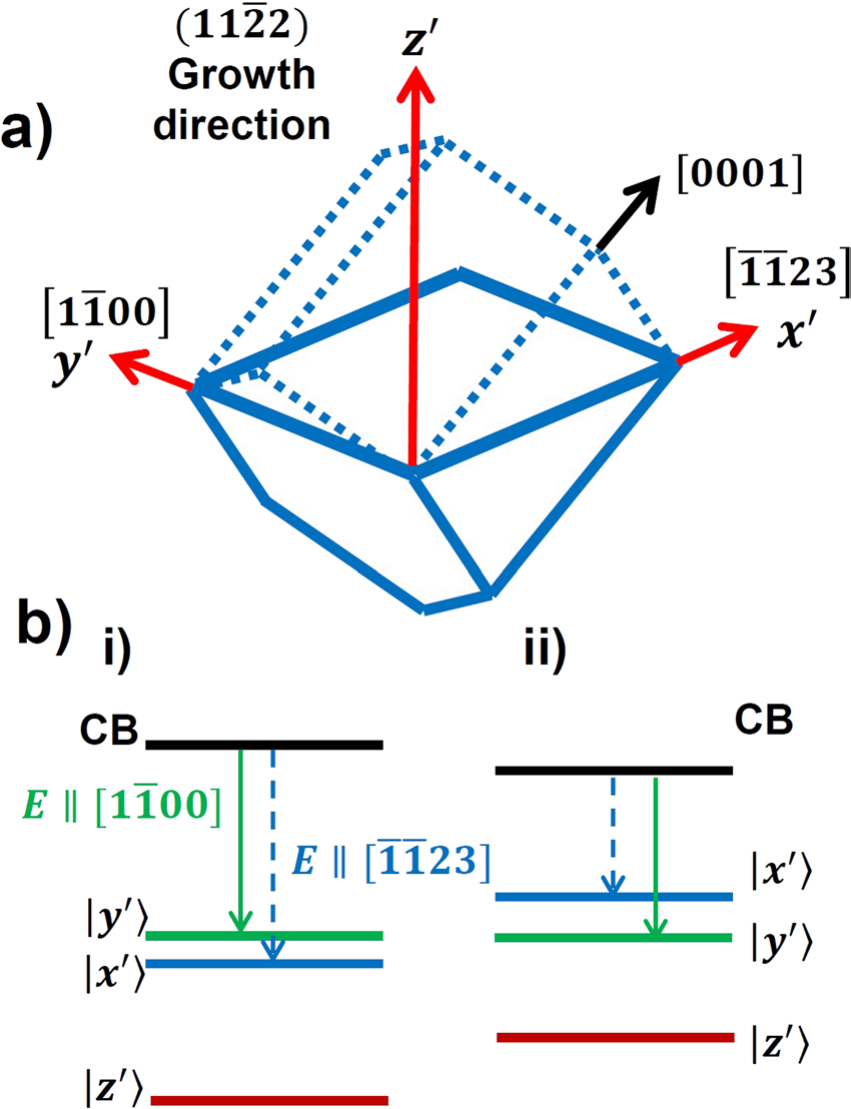
Schematics of (**a**) semi-polar (11–22) GaN crystal plane and (**b**) band diagram before and after polarization switching.

Figure 1a schematically illustrates a primed coordinate system showing that the $x{\prime}$ - $y{\prime}$ plane represent the (11–22) GaN plane, where the two existing orthogonal directions are labeled by $x{\prime}$ and $y{\prime}$. $x{\prime}$ represents the [−1–123] direction that is parallel to the projection of the c-axis $(\Vert c{\prime} )$, where $y{\prime}$ shows the [1–100] direction perpendicular to this c-axis ($\perp c$), and $\,z{\prime}$represents the growth direction. Owing to anisotropic strain the valence subbands of the (11–22) GaN split into $|y{\rm{{\prime} }} >$ and $|x{\rm{{\prime} }} >$, where $|y{\rm{{\prime} }} >$ and $|x{\rm{{\prime} }} >$ are the first and the second valence subbands, associated with the respective emission from dipoles along the [1–100] and [−1–123] directions. However, with increasing indium content these two otherwise separate valence subbands approach each other and eventually exchange positions. As a result, the highest valence subband becomes $|x{\rm{{\prime} }} >$ with a dipole parallel to [−1–123], while the second valence subband then becomes $|y{\rm{{\prime} }} >$ with a dipole parallel to [1–100]. Figure 1b provides a schematic illustration depicting this change before (i) and after (ii) polarization switching.

A polarization degree, denoted *ρ*, is defined by the ratio of the integrated polarized emission intensities along one direction relative to another direction as expressed below:

1 $$\rho =\frac{{I}_{[1\bar{1}00]}-{I}_{[\bar{1}\bar{1}23]}}{{I}_{[1\bar{1}00]}+{I}_{[\bar{1}\bar{1}23]}}$$

A positive polarization ratio (*ρ* > 0) means that the dominant emission component is polarized along the [1–100] direction, while a negative polarization (*ρ* < 0) demonstrates that the dominant polarization component is along the [−1–123] direction.

Electroluminescence (EL) measurements have been carried out at room temperature in a continuous wave (cw) mode. A detailed description for the EL measurement system has been provided in the “Methods” section, and a schematic for the system has been illustrated in Fig. 3S in Supplementary Information. There is a rotating linear polarizer placed between an objective lens and spectrometer, which allows polarization dependent EL measurements to be conducted between the two orthogonal directions, namely [1–100] and [−1–123]. The 0° and 90° angles of the polarizer position correspond to the electric fields aligned along [1–100] and [−1–123] directions, respectively.

**Figure 2:**
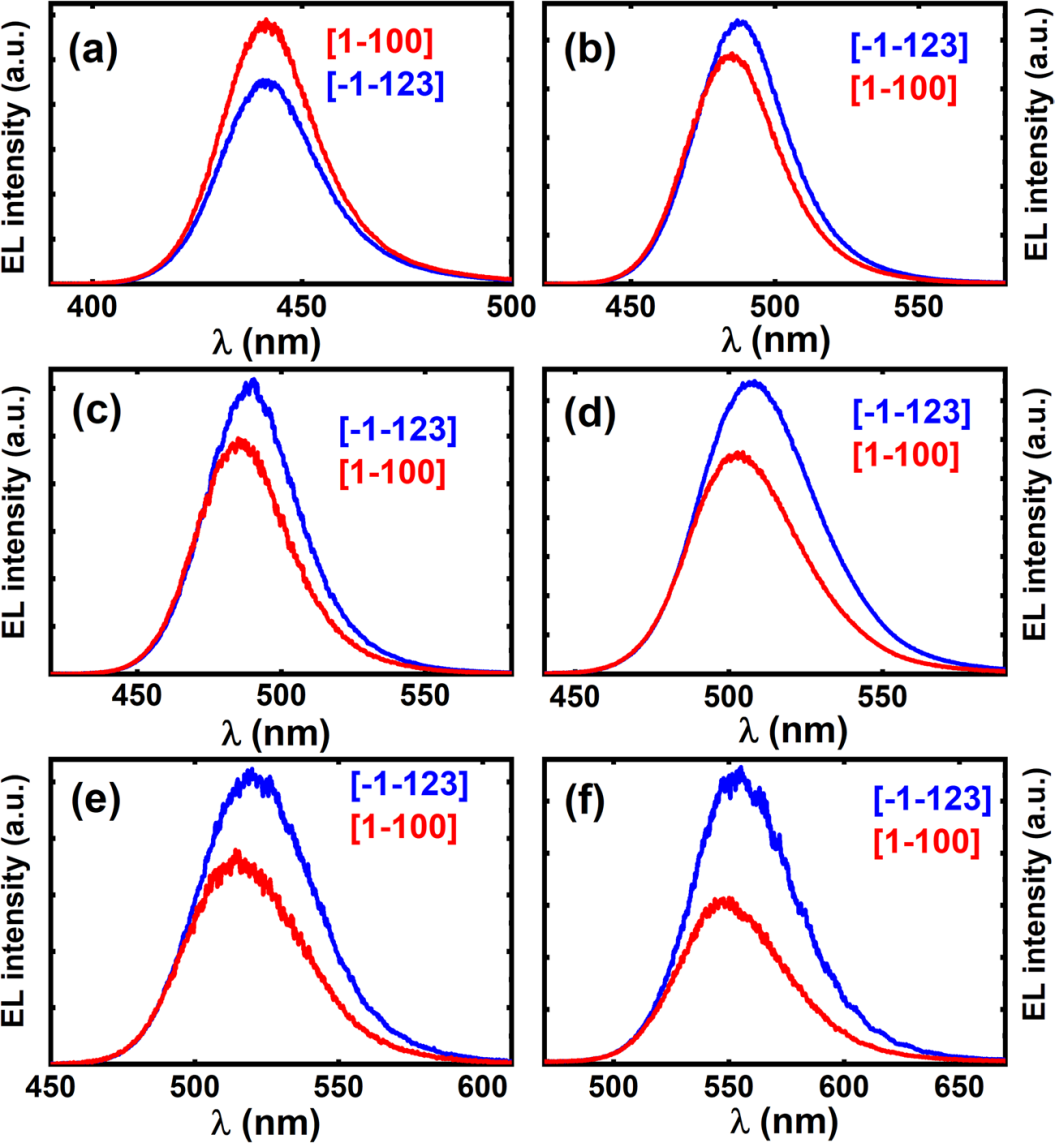
Polarized EL spectra measured at 20 mA injection current for the semi-polar (11–22) LEDs with increasing indium content and peak emission wavelength. In each case, the EL spectrum labeled in red was measured at a polarizer angle of 0° corresponding to the electric field aligned along [1–100], while the EL spectrum in blue was measured at a polarizer angle of 90° corresponding to the electric field aligned along [−1–123].

Figure 2 shows the EL spectra of each of the semi-polar InGaN LEDs with a peak emission wavelength ranging from blue (443 nm) to yellow (555 nm), which are measured with the polarizer aligned at 0° and 90° under 20 mA injection current. In each case, the EL spectrum colored with a red-line was measured with the polarizer positioned at a 0° angle, corresponding to the electric field aligned along [1–100] direction, while the EL spectrum labeled with a blue-line was measured at a 90° polarizer angle, indicating the electric field aligned to the [−1–123] direction. This demonstrates that the polarized EL spectra depend on the peak emission wavelength and therefore indium content.

**Figure 3:**
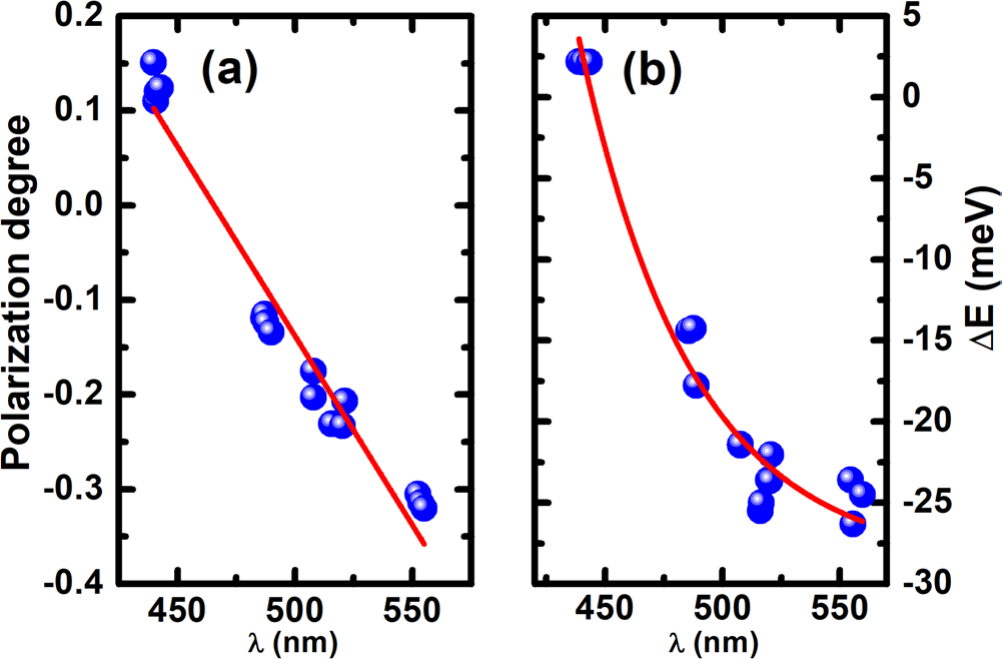
(**a**) Polarization degree as a function of peak emission wavelength; and (**b**) Energy separation Δ_Ε_ of the two polarized emissions as a function of peak emission wavelength. Solid lines are only as a guide to the eye.

Figure 3a shows the polarization degrees of all the semi-polar LEDs as a function of peak emission wavelength, where the polarization degree has been extracted from Fig. 2 using Eq. 1.

Figure 3a shows a polarization degree ranging from 0.15 for the shortest wavelength emitter (443 nm LED) to −0.33 for the longest wavelength LED (555 nm LED). The polarization degree of the 443 nm blue LED exhibits a positive sign (*ρ* > 0), meaning that the intensity of the emission with the electric field polarized along the [1–100] direction is higher than that of the emission with the electric field polarized along the [−1–123] direction. Figure 3a also demonstrates that the polarization degree approaches zero at ~470 nm. For the longer wavelength LEDs above ~470 nm the polarization degree therefore switches to a negative sign (*ρ* < 0) which signifies that the intensity of the polarized emission along the [−1–123] direction is now higher than that of the polarized emission along the [1–100] direction. This is entirely consistent with previous studies^19,25,26^. As the emission moves towards longer wavelength, the polarization degree becomes larger in the negative direction owing to the larger energy separation of the two topmost $|x{\rm{{\prime} }} >$ and $|y{\rm{{\prime} }} >$ valence subbands.

Figure 3b depicts the energy separation between the two polarized emissions (along [−1–123] and [1–100] directions) for each LED as a function of peak emission wavelength. The energy separation labeled as ${\Delta }_{E}$ can be defined as:

2 $${\Delta }_{E}={E}_{P[\bar{1}\bar{1}23]}-{E}_{P[1\bar{1}00]}$$

where ${E}_{P[1\bar{1}00]}$ and ${E}_{P[\bar{1}\bar{1}23]}$ correspond to the peak energies of their emission with the electric field polarized along the [1–100] and [−1–123] directions, respectively.

Figure 3b shows that the ${\Delta }_{E}$ between the two polarized emissions of the 443 nm blue LED is positive. As the emission wavelength increases, the ${\Delta }_{E}$ becomes negative and continues to further reduce with wavelength meaning that the absolute value of ${\Delta }_{E}$ further increases. A comparison between Fig. 3a,b depicts that a negative polarization degree (*ρ* < 0) is always connected with a negative valued energy separation (Δ_Ε_ < 0), which is in agreement with other report^19^. This is due to the transitional probability between the topmost valence subband and the conduction band being higher than that between the second topmost valence subband and the conduction band.

**Figure 4:**
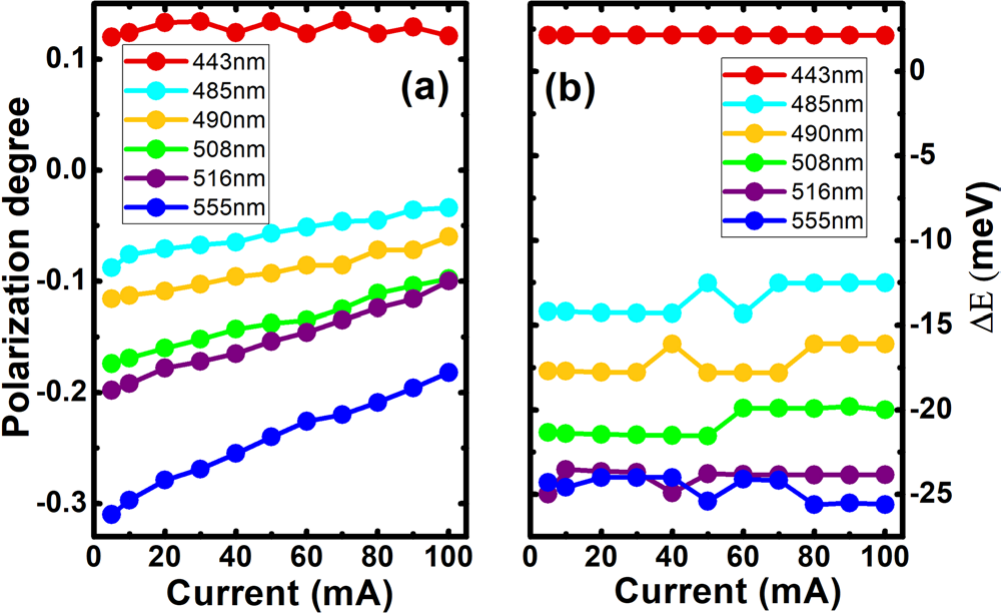
(**a**) Polarization degree as a function of injection current; and (**b**) Energy separation Δ_Ε_ as a function of injection current for each of the semi-polar LEDs.

Figure 4a depicts the polarization degrees of all the LEDs measured as a function of injection current from 5 to 100 mA, while Fig. 4b shows the Δ_Ε_ between the two polarized emissions (along [−1–123] and [1–100] directions) also as a function of injection current in the same range. It is noted that the 443 nm LED exhibits a different relationship to polarization degree (*ρ*) and Δ_Ε_ with injection current in comparison to the longer emission wavelength LEDs. The longer wavelength LEDs with a negative polarization degree (*ρ* <0) demonstrate that an absolute value of polarization degree (|*ρ*|) decreases with increasing injection current, while both the polarization degree and the Δ_Ε_ of the 443 nm blue LED remains almost constant regardless of injection current. Furthermore, for each of the longer wavelength LEDs, the change in polarization degree decreases with increasing injection current in each case. In addition, this change in polarization degree also increases with increasing indium content (emission wavelength). In greater detail, the change in polarization degree from 5 to 100 mA is approximately 0.06, 0.1 and 0.14 for each of the 485, 508 and 555 nm LEDs, respectively. Figure 4S in Supplementary Information also provides very clear data.

The reduction in the absolute value of polarization degree with increasing injection current can be attributed to band filling effects. In the longer wavelength LEDs, the topmost valence subband is $|x{\rm{{\prime} }} >$ with a resulting dominant emission component polarized along the [−1–123] direction, while an emission associated with the second valence subband $|y{\rm{{\prime} }} >$ is polarized along the [1–100] direction. At low injection current holes mainly occupy the first valence subband $|x{\rm{{\prime} }} >$. With increasing injection current holes then begin to fill the second valence subband $|y{\rm{{\prime} }} >$ states, once the more favorable states of the first subband are fully occupied. Consequently, the $|y{\rm{{\prime} }} >$ valence subband related emission increases with increasing injection current, and therefore the overall polarization degree decreases.

However, the blue 443 nm LED demonstrates a polarization degree that remains nearly constant with increasing injection current, which is similar to an existing report^27^. This effect is attributed to the significantly higher density of states (DOS) associated with the $|y{\rm{{\prime} }} >$ subband (polarized emission along [1–100] direction) than is associated with the $|x{\rm{{\prime} }} >$ subband (polarized emission along [−1–123] direction^25^. Consequently, the $|y{\rm{{\prime} }} >$ subband can accommodate a significantly high density of holes, leading to a lower probability for holes to occupy the $|x{\rm{{\prime} }} >$ subband before polarization switching. This naturally means that the polarization degree remains unchanged with increasing injection current.

In principle, Δ_Ε_ is determined mainly by the difference in energy state between the $|y{\rm{{\prime} }} >$ and $|x{\rm{{\prime} }} >$ subbands, and thus is not sensitive to injection current. Consequently, as shown in Fig. 4b, the Δ_Ε_ remains almost unchanged with increasing injection current.

In conclusion, a systematic study of the influence of both indium content and injection current on polarization properties has been performed on a series of semi-polar LEDs with a wide spectral range between 443 and 555 nm all grown on (11–22) semi-polar GaN templates with a high crystal quality. Detailed polarization dependent EL measurements demonstrate that the polarization degree strongly depends on the LED emission wavelength, which varies from a positive polarization degree of 0.15 at the shortest wavelength (443 nm) to a negative polarization degree of −0.33 for the longest wavelength LED. A linear fitting indicates that a polarization switching takes place at around 470 nm. Furthermore, the longer wavelength LEDs with a negative polarization degree exhibit a consistent relationship between polarization degree and injection current, while the 443 nm blue LED (before polarization switching) exhibits an insensitivity in polarization degree to injection current.

## Methods

### Epitaxial growth

All the semi-polar LEDs were grown on high quality (11–22) semi-polar GaN templates on *m-plane* sapphire by a low-pressure metal-organic vapour phase epitaxy (MOVPE) system. The semi-polar (11–22) GaN templates were obtained by using our well-established overgrowth approach on micro-rod arrays, where the micro-rod diameter is typically 4 µm^11–13^. For the micro-rod array fabrication, a SiO_2_ layer with a thickness of 500 nm was initially deposited on a standard single semi-polar (11–22) GaN layer with a thickness of ~400 nm grown on *m-plane* sapphire. Subsequently a standard photolithography patterning technique and then dry-etching processes were employed to etch the SiO_2_ film into regularly arrayed micro-rods with a diameter of 4 µm, which serve as a secondary mask to etch GaN underneath, forming regularly arrayed GaN micro-rods. Finally, the regularly arrayed semi-polar GaN micro-rods were reloaded into the MOVPE chamber for overgrowth. The overgrown semi-polar (11–22) GaN layer with a thickness of ~4 μm exhibits a typical dislocation density of 2×10^8^ cm^−2^ and a typical basal stacking fault density of 4 × 10^4^ cm^−1 ^^11–13^. All the LED structures were further grown on the semi-polar overgrown templates, beginning with a 1 μm Si-doped *n*-type GaN layer, then InGaN/GaN SQW and a final 150 nm Mg-doped *p*-type GaN layer. The only difference in the growth between these LEDs is the growth temperature for the InGaN SQW used to control the indium content, allowing for emission wavelengths ranging from 443 to 555 nm.

### Device fabrication

By means of a standard photolithography technique and subsequent dry-etching processes, LEDs with a standard mesa size of 330 ×330 μm^2^ have been fabricated. 100 nm ITO layer was used as a transparent *p*-type contact, while an *n*-type Ti/Al/Ti/Au alloy contact was then deposited on to *n*-type GaN. Finally, Ti/Au alloy bond-pad are deposited by thermal evaporation to form both *p*-type and *n*-type contact electrodes.

### Electroluminescence (EL) measurements

Polarization dependent EL measurements have been conducted by using an electroluminescent system equipped with A Keithley 2400 Source-meter and an objective lens (50× magnification; NA = 0.42). EL emission is collected by the objective lens, and is directed through a 50:50 beam splitter. 50% of the emission goes to a CMOS camera which is used to identify the position of a sample. The rest 50% emission is then introduced into the Shamorck 500i Czerny-Turner monochromator via a fiber collimator and finally detected by an air-cooled charge coupled device (CCD). A rotatable polarizer is placed between the objective lens and the fiber collimator, allowing for polarized EL measurements. A halogen lamp is used as a calibration light source in order to remove any polarization from the system. (refer to Fig. 3S in Supplementary Information for a schematic diagram for the EL system).

## Supplementary information

Supplementary Information.
