# Supplementary material for: Optical polarization properties of (11–22) semi-polar InGaN LEDs with a wide spectral range
Source: Sci Rep. 2020 Apr 28;10:7191. doi: 10.1038/s41598-020-64196-w (PMC7188879; doi:10.1038/s41598-020-64196-w)

Optical polarization properties of (11-22) semi-polar InGaN LEDs with a wide spectral range

N. Poyiatzis, J. Bai, R. M. Smith, M. Athanasiou, S. Ghataora and T. Wang*

Department of Electronic and Electrical Engineering, University of Sheffield, Mappin Street, Sheffield S1 3JD, United Kingdom

*E-mail: t.wang@sheffield.ac.uk

Supplementary Information

Figure S1: Typical cross-sectional TEM image of our (11-22) LED overgrown on a micro-rod arrayed template (a) and Typical plane-view TEM image of our (11-22) LED structure, showing that the dislocation density is 2.0×10^8/cm^2. For detailed structural characterisation, please refer to Refs.11-13.

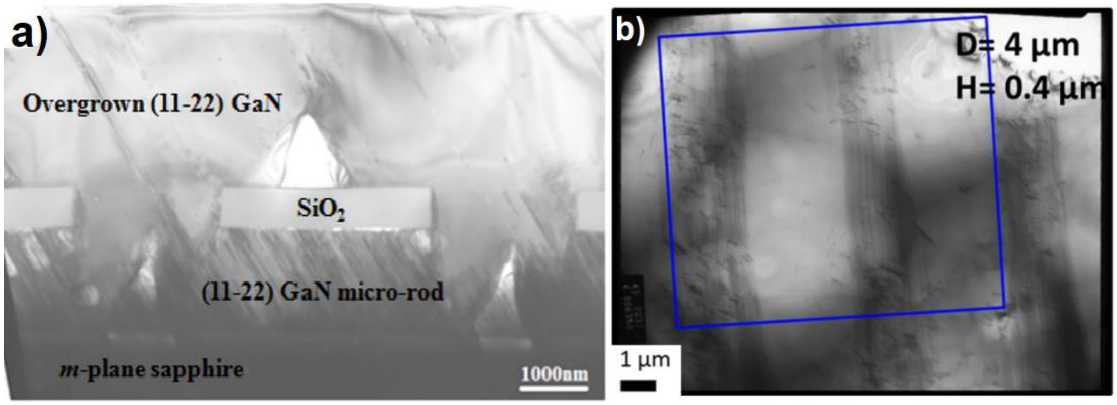

Figure S2: Schematic illustration of our semi-polar (11-22) InGaN LEDs.

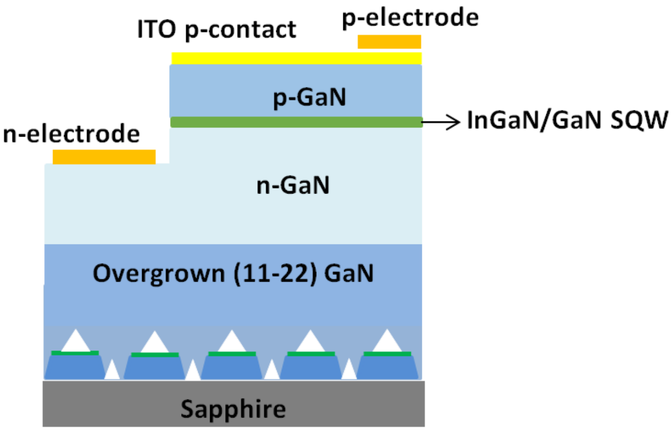

Figure S3: Schematic diagram of our electroluminescence measurement system (A detailed description is given in the “Methods” section in the paper)

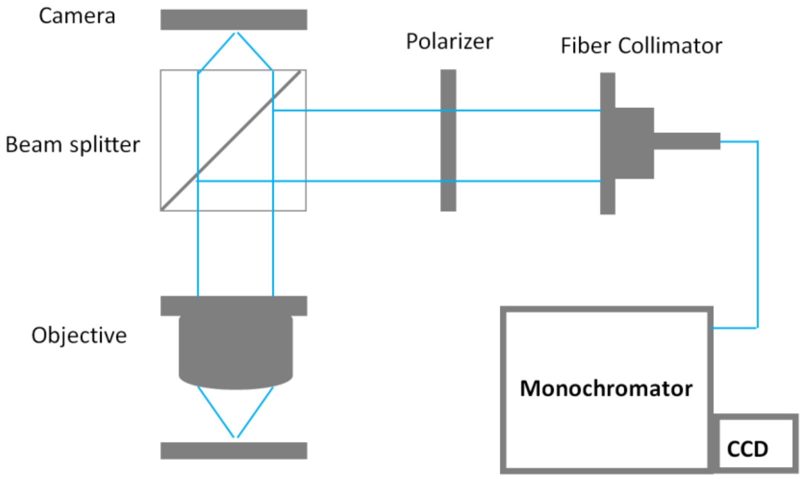

Figure S4 shows the change in polarization degree as a function of emission wavelength, exhibiting that it increases with increasing emission wavelength. This further supports the conclusion explained by using the band-filling effect mentioned in the paper.

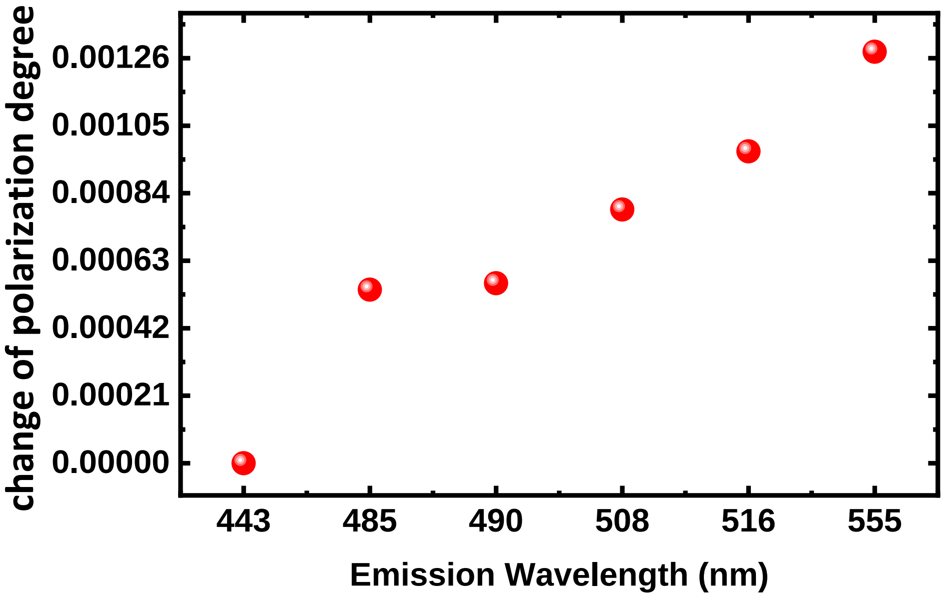

Supplement: Supplementary file 1 — Supplementary Information. [file 41598_2020_64196_MOESM1_ESM.pdf]
